# The context of care as a supporting axis for comfort in a palliative care unit

**DOI:** 10.1177/26323524241258781

**Published:** 2024-06-12

**Authors:** Raquel Alexandra Machado Pereira, Patrícia Cruz Pontífice Sousa Valente Ribeiro

**Affiliations:** Universidade Católica de Lisboa, Palma de Cima, Edificio Reitoria, Lisboa 1649-023, Portugal; Universidade Católica de Lisboa, Lisboa, Portugal

**Keywords:** comfort, context of care, ethnography, palliative care

## Abstract

**Background:**

The context of care determines and organizes practices through its structures and guiding principles. It is sometimes a space that generates tension and multiple choices, variable in the provision of different care and uncertain in its duration. We can consider that the construction of the comfort process does not only depend on the will of its actors and the situation itself, but is also conditioned by the professional, cultural, and social context in which it is inserted. This article is part of a doctoral study in the field of comfort in a palliative care unit, and these are some of the partial results that emerged.

**Design:**

Qualitative study using ethnographic approach.

**Methods:**

We conducted semistructured interviews with 18 patients at the end of life and their matched significant family members (18) and 21 health professionals. We also conducted a participant observation of care situations.

**Results/discussion:**

The context of action, where meanings and practices are learned, is linked to a certain identity that is related to practical, contextual knowledge, linked to a collective and to a feeling of belonging. The relationship between the various factors that shape the Care Context in the palliative care unit studied, constitute the three domains of this topic, specifically: *the integrative and inclusive environment, the conceptions of care*, and *the inclusive factors of organizational culture.*

**Conclusion:**

The specific context was determinant as a supporting axis for comfort in this palliative care unit. The context of care, where objects and provisions support the construction of the comfort process as an entity that integrates culture, established conceptions of care, allowing the deepening of knowledge.

## Introduction

Comfort is an important concept and of enormous value for the nursing profession^1,2^ constituting a key element in the provision of care to the person at the end of life. The process of living has been exponentially extended in recent decades, due to technological innovations and their impact on increasing survival, which makes us realize that the end of life, in most cases, is no longer an episode but a process that can take years or even decades depending on the type of disease we are dealing with. It is now internationally recognized that, when applied early, Palliative Care (PC) brings benefits to patients and their families, reducing the symptomatic burden of patients and the burden on family members. Furthermore, PC reduces hospitalization, readmissions, therapeutic futility, the use of emergency services and intensive care and, consequently, reduces healthcare costs. PC units refer to qualified and properly organized services, where professionals with training in PC work with experience in caring for people at the end of life, accompanying them 24 h a day, developing holistic care, dedicating themselves exclusively to provide this specialized care, and following this care philosophy in their practice.^3^

In Portugal, currently, most people spend their last days of life in hospitals, but only some of these people spend those days in a Palliative Care Unit (PCU). Given the complexity of the problem faced by people at the end of life, it is essential to reflect on the practice of care, in order to identify existing knowledge and the need to develop and mobilize new knowledge and skills to improve the provision of care. We are dealing with a person with a serious/progressive/incurable illness, with physical, psychological, social, and spiritual needs, which require specific care provided by a multidisciplinary team following a holistic approach. The objective of PC is to provide maximum comfort, promoting maximum quality and dignity of life for the patient and family, without the intention of accelerating or delaying the death process.^4–7^

Comfort transcends the physical dimension, it is significantly more than the absence of pain, and it involves physical, psychospiritual, social, and environmental components. Therefore, palliative teams must act to promote comfort in all its dimensions. Through a multitude of contextual aspects, nurses and other health professionals seek to carry out the complex activity of accompanying human beings at the end of life, helping them to die with dignity and serenity. However, in hospitals, medical values related to the cure of the disease prevail, and the working rhythms are very accelerated. It is a real challenge to implement in these institutions models of care for people at the end of life that are based on promoting quality of life, in which comfort and well-being take priority, to the detriment of more invasive measures. The study context, a PCU, where the construction of comfort as an entity that integrates culture takes place, arises from the specific environment of the service and the characteristics and actions constructed by the group of actors and their dynamics. We wanted to understand how comfort is built and developed in this unit. To this end, it was essential to understand the context where the meeting with the actors took place, observing details related to the space itself, the care provided and the interactions established. The context of care is determinant to the processes. We can consider that the construction of the comfort process does not only depend on the will of its actors and the situation itself, but is also conditioned by the professional, cultural, and social context in which it operates. The social space where the action takes place, involves physical behavior and also the meanings attributed to it, as a whole.^8^ In this logic, the context of action, where meanings and practices are apprehended, is linked to a certain identity that is related to practical and contextual knowledge as well as a feeling of belonging.^9^

The replication of studies on comfort in different contexts allows the emergence of new contributions to practice, in this case in the area of people at the end of life, who, as we have seen, are in great need of healthcare. It contributed to strengthening not only nursing interventions but also palliative team interventions. We are talking about informants, patients who participated in the study who have a high degree of vulnerability. The contribution of the study is, for them, toward the creation of this model of care organization. Furthermore, it is a study that gives us the perspective of the various groups of actors, which is new knowledge.

Accompanying the suffering of the dying person is an act of love and commitment that is assumed by professionals, but should not be assumed only by health professionals. It must be assumed by care-providing organizations. This study uncovered that the comfort relationship we observed in this micro culture is established in the interaction between conditions (structure), which is the responsibility of the institutions; the actions (the process) and the consequences (results).

Therefore, we believe that it will always be useful to explore knowledge in the development of the profession as it is a phenomenon of interest relating to human responses in the health-illness journey, an integrative concept, which includes different indicators of people’s satisfaction and, which can be evaluated, seeking to promote evidence-based care organization models, in contexts similar to the one we studied.

## Methods

### Adopted definitions and framework

This is a qualitative study using an ethnographic approach. Among the qualitative research approaches used in nursing and in the field of health to obtain data related to the culture of a group and the way in which health professionals exercise their autonomy in their daily work, ethnography and ethno-nursing stand out.^9,10^ These methods are powerful ways of obtaining ‘facts, feelings, worldviews and other types of data that reveal the real world, truths and people’s ways of life’, allowing the understanding of beliefs and values.^10^ We followed the model proposed by James Spradley,^11,12^ and we have chosen this method because it allows us to make sense of the lived experience, the way in which the situation is culturally experienced, interpreting it in its natural context from the different points of view of the participants to obtain meaningful contents that allow describing and understanding human experiences.

The reporting of this study conforms to the Equator Network Guideline COREQ^13^ (Supplemental File).

### Methodological procedures

Research based on ethnography allows one to look beyond the evidence, looking for the underlying and implicit meanings that people, as members of a subculture, attach to their practice. What interests the ethnographer are the common experiences lived in a group endowed with a culture in which the human being (person at the end of life, family members, and caregivers) is seen as someone who interacts with himself, with others and with the environment.^14^

As foreseen in ethnography, we used several data collection methods that complement each other. We started with participant observation, as it allows us to understand the consonance between what people say they do and what they actually do because, sometimes, people are not aware of the subtleties of what happens in the interactions between them.

Participant observation was carried out by the ethnographer, in interaction with the informants throughout their daily activities and in this way the researcher, as an external observer, produced reports of the observations carried out, through recording notes, field diary, photographs and/or recordings. The interactions that arise from this led to reflection and, for this purpose, we used a reflective diary, as suggested by Spradley.

Initially, before moving into the field, we created a flexible observation guide, a guiding thread for our observation. Therefore, we created the observation guide base on the research question, the objectives of the study, the notes from the participant observation, and the literature we had consulted to develop the project’s issues. In a second moment, we carried out an observation with some participation, where we encouraged interaction with the actors. Subsequently, we planned participation with some observation where the researcher became an active participant in the informants’ activities. At this stage, some interviews also began to take place.

The observation thus progressed, being planned and focused on a concrete objective, determining the aspects, places, and people to observe. Understanding how comfort is constructed, as an entity that integrates culture in a PCU, involves identifying the specificities of this same process, making it necessary to study and understand the organization in general, the actors in their context as well as the meaning of their actions. As we progressed, our records became more structured, differentiating the language of the researcher and actors according to the coding determined.

We also carried out semistructured in-person interviews with all the participants: people at the end of life, family, and health professionals, based on a script that was submitted to a pre-test with one patient, one health professional, and one family member who were not part of the participants in our study. With this, we aimed to test the clarity and understanding of the questions included in the interview.

### Study setting

Based on the purpose of the research, a PCU of a hospital on the outskirts of Lisbon was chosen as the setting for the study. The choice of location was intentional and based on practical and methodological reasons: being geographically accessible; being a PCU integrated within a hospital center, with people at the end of life, the vast majority in need of symptom control, whether physical or emotional; and finally because this particular unit is one of the few in Portugal that integrates a multidisciplinary team made up of doctors (including a physiatrist), nurses, operational assistants, superior social service technician, therapists, psychologist, dietician, and spiritual assistant.

The PCU integrates the following areas in an integrated manner:

○ *Inpatient care:* Has eight inpatient beds, distributed across a double room and six single rooms, for patients requiring acute PC;
○ *Hospital outpatient clinic:* To provide PC on an outpatient basis;
○ *Consultancy/intra-hospital team:* To support patients admitted to other adult services at the Hospital Center.

This PC unit chosen for our study is in excellent condition as it is a relatively new service (opened 12 years ago) and consists of: eight inpatient beds, distributed across a double room and six single rooms, for patients requiring acute PC, each with its own bathroom for use by patients; one medical office, one nurse manager office; two bathrooms for the staff; one patient dining room/reading room/family conference room; one nursing office/station where medication is prepared and some materials are stored; a central desk with two computers; one shift change office; one kitchen; one assisted shower room; one clinical material storage room (management of material resources based on a picking system); and one treatment room. At the end of the corridor, there is a fire door that gives access to the emergency stairs, which, as a general rule, is not used by service professionals.

**Figure 1.**
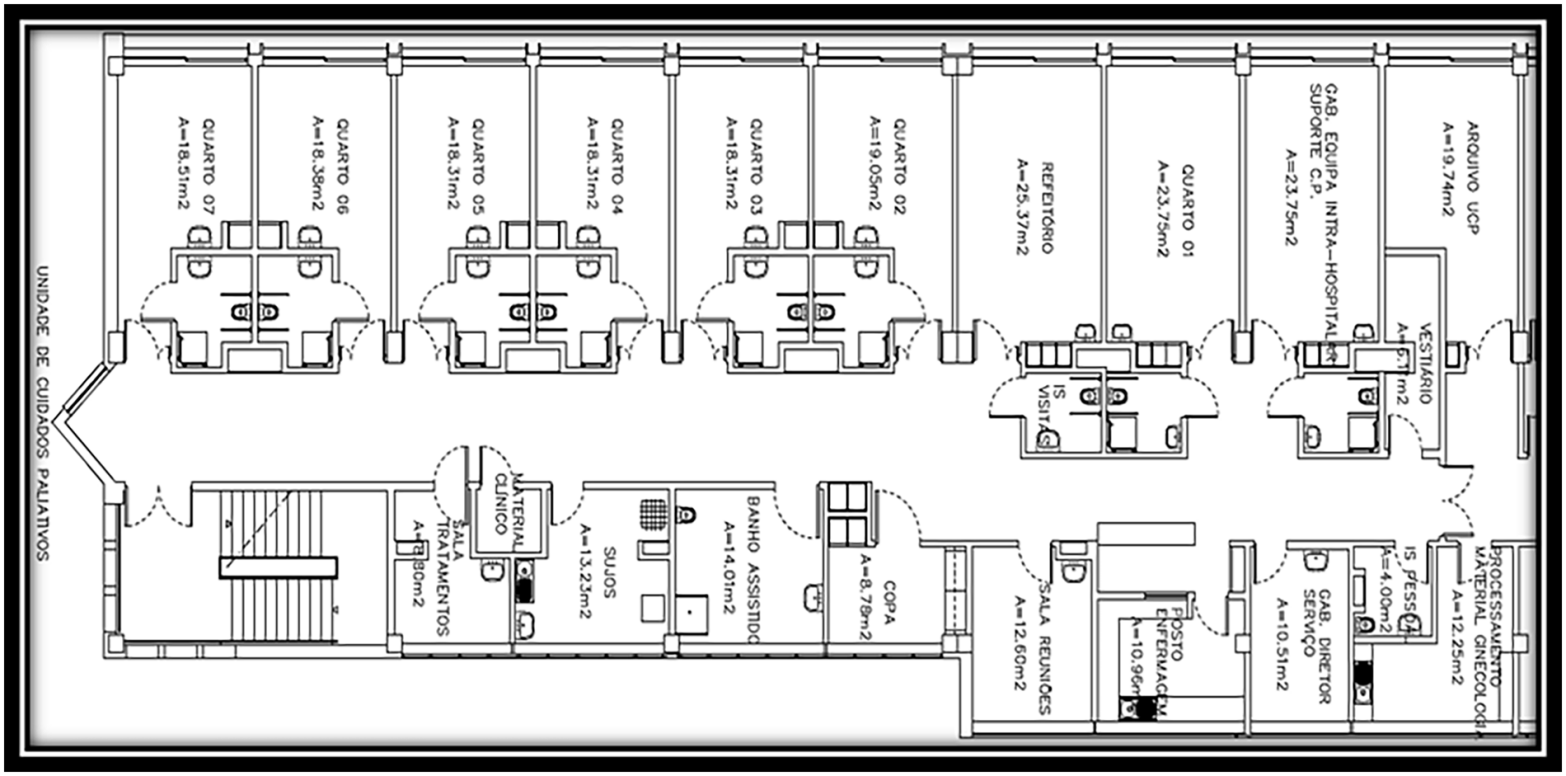
Plan of the UCP hospitalization.

The complete plant of the PC unit acute inpatient area can be seen in Figure 1.

Physical spaces are of great importance as a source of data in understanding the social and cultural relationships between the different professionals who interact, which is why we described some in more detail.

To define the participants, for the sample selection, we defined the following inclusion criteria: being hospitalized in the acute care unit with an incurable, chronic disease that causes suffering; be aware and oriented in order to be able to respond orally to the applied questions; freely consent to participate in the study. For each patient interviewed, their significant family member was also interviewed. The selection of people at the end of life and family members were carried out by the main researcher, based on prior knowledge of hospitalized people and information obtained from nurses and other professionals, or by consulting data in clinical files. We had to make sure that patient and family member both wanted to participate in the study before the interviews, if one of them did not accept to participate, they would both be excluded from the sample. Finally, all healthcare professionals in the unit were interviewed after consent.

With regard to observation, we sought access to the field and paid attention to the experiences lived by the actors in the events and situations that were taking place over time.

### Participants

The actors involved in our study include a total of 57 participants among people at the end of life, professionals, and family members: 18 patients at the end of life; 18 matched family members; and 21 health professionals. It was absolutely necessary that patient and family member accept to participate since we intended to ask questions related to the situation to both of them and then compare the answers. We also gave some information on the interviewer to the participants: nurse with major interest in PC who was conducting a doctoral study on the subject.

The selection was carried out by the researchers, with the collaboration of the nurses present in the respective shifts, taking care to verify the availability of each patient through an initial presentation dialogue. Regarding the state of consciousness and the ability to respond orally, in the presence of doubts, we resorted to the help of nurses and also, we used the Glasgow Coma Scale to assess orientation and ability to answer questions effectively.

The final composition of the sample was determined by data saturation, as well as the achievement of information redundancy, in which new information came to confirm the previous ones, not objectively adding new data.^15^

There was no previous contact/relation between the researchers and the staff unit. The first author/main researcher who conducted the interviews was a female, RN, MSc.

### Data collection methods

Data collection was carried out between October 2019 and January 2021, for a total of about 16 months in interactions, in order to fulfill the participant observation phases and interview all informants. A total of about 137 h were spent in the field, resulting in 78 field notes and 57 recorded interviews (all participants were observed and interviewed: patients, family members, and health professionals). During this period, we stayed in the field 1–5 h/day, in scattered periods, distributed in the three shifts and at different times of the day, as we considered important to observe the participants at different times. However, we favored the morning and afternoon shifts as they have a larger number of participants on the ground at the same time, and it is easier to observe their interactions.

With regard to participant observation, before moving into the field, we prepared a flexible observation guide for our observation, open and wide enough not to focus only on what it anticipates, but also on everything that may arise around it and that is of interest for the study, even if it is not included in the guide. This way, very general aspects to be observed were foreseen, which refer to what Spradley^11^ calls ‘descriptive observation’. It was intended to involve the researcher in the context of the PCU to get to know all the actors in it, observing the participants and allowing them to become familiar with the researcher (we observed all the activities of the staff, the patients and families: conversations, family conferences, meals, interactions, and shift changes, among others). In a second moment, we carried out an observation with some participation, we encouraged interaction with the participants by recording their reactions, and the focus became the people involved in the context. Subsequently, a participation with some observation was planned where the researcher became an active participant in the activities of the informants.^11^ All of this information was registered in a field diary resulting in 78 field notes. The observation and the interviews were done simultaneously due to the data collection method.

The interviews were audio-recorded in Portuguese, transcribed, and the data collection instrument included questions related to the characterization of the participants and open-ended questions related to representative situations of comfort care, namely, comfort-promoting strategies mobilized by health professionals and particular moments of comfort and discomfort. Prior to the interview, the location, time, and date were agreed on. When interviewing professionals, we took into account their occupations, so that we would not interfere in the care of people hospitalized in the unit. When interviewing patients, the interviews were carried out either in the patient’s own unit or in a private environment. During the interview, the main researcher tried to make it flow spontaneously, following the flow of the interviewee’s ideas. We previously carried out the legitimization, clarifying doubts, and requesting authorization to turn on the recorder. All interviews were coded, based on the professional category and the number we attributed to it. The time of each interview was variable; between 30 and 45 min for health professionals and between 40 and 60 min for the person at the end of life and the family, this variation in time being due to the fact that patients and families are not conditioned in terms of time. We organize a categorization system with all the data, this being a work of discovery, with the objective of selecting and organizing the data for further analysis.

### Data analysis

In ethnography, data analysis begins when data collection begins and constitutes a cyclical, reflective, systematic, and integrated process, often implying a constant reformulation of questions, thus accompanying the entire investigation process.^11,12^ This whole process implies and involves analysis, thought and reflection, carried out around a systematic examination in order to find the parts and the relationships established with the whole.^11,12^ It also allows reiterating sustained cultural patterns in the description of cultural behaviors, artifacts, and cultural knowledge.

As we collected the data, we proceeded to organize and systematize it in order to proceed with its codification, systematic comparison, and analysis to find similarities and differences, with a view to defining phrases that could be constituted of meaning. We tried to carry out a progressive analysis that allowed the discovery of beliefs, values, and practices that underlie the culture of care. To meet these standards and subsequently the domains, categories, subcategories, and sub-subcategories, we analyzed various types of data, from the field diary and unit documents (medical and nursing records, specific notes about comfort in the clinical process of patients), as well as data from interviews.

Continuing the analysis, the guiding principle was to meet the recommendations of Spradley: we reviewed and identified domains based on the information we had; observed similarities and relationships between concepts, which we incorporated into categories and subcategories, which we reorganized; identified new categories, subcategories, and sub-subcategories that led us to the reflection recommended by Spradley on ethnographic detail; created broader domains that included previously analyzed structural issues and data from all sources; resorted to the NVivo 12 Plus program; and started to organize, articulating categories and subcategories. This application made it possible to store and organize the different sources, categorize, and classify qualitative data. This choice also allowed us to easily cross data, map ideas, and explore relationships between the actors.

### Methodological rigor

A research question must be clear and focused and supported by a strong conceptual framework, both of which contribute to the selection of appropriate research methods that enhance trustworthiness and minimize researcher bias inherent in qualitative methodologies.^16^ Qualitative data collection and analyses are often modified through an iterative approach to answering the research question. Researcher reflexivity, essentially a researcher’s insight into their own biases and rationale for decision making as the study progresses, is critical to rigor.^16^ The analysis of the data itself was carried taking into account the above, by having checks and balance built into the methodology.

The work supervisor and another researcher with knowledge of the ethnographic method, added a non-bias approach. The *corpus* (composed of the field diary and all the interviews carried out) was read and revisited repeatedly throughout the entire analysis by the included researchers. We wanted to follow a scientific and rigorous method that allowed the organization, division, synthesis, and search for some patterns in the data to be analyzed independently, guaranteeing a varied and comprehensive analysis, not conditioned to the understanding of a single researcher. Comparison sessions of data analysis were carried out by the three researchers involved, ensuring the necessary triangulation in an analysis of this nature.

With regard to the topic, the use of various participants and different perspectives (person at the end of life, family, and professionals) was also an important tool to ensure *credibility.* During the fieldwork, we sought to validate with the informants the contributions we were collecting, trying to understand whether our results were, in fact, meeting their experiences.

In order to overcome the methodological limitations, in addition to what was already mentioned after the analysis, we discussed the results with the unit’s coordinating nurse and with the team of professionals, since they could observe what Leininger alluded to as the *meaning in context*.^17^ These actors either knew the study, helping with the analysis of the relationship between domains and categories, or knew the context, which allowed the discussion of results, in order to understand if they were logical and legitimate. On the other hand, we observed *saturation* and only finished collecting data when we found repeated patterns (with regard to patients and family members, since all professionals were interviewed). *Transferability* was also observed, carrying out rigorous reports in order to allow the transfer of knowledge based on the results, to other care situations that have similar cultural conditions. It should also be noted the conversations/sharing of information with other researchers about the methodological route, the results and the bibliography in an attempt to consolidate *fidelity*. This sharing allowed the crossing of routes and results with other members of the scientific community besides the researchers.

## Results/discussion

There were 57 participants in the study, including patients, families, and professionals. The sample consisted of 18 patients at the end of life; 18 family members (for each patient, the matched significant family member were interviewed) and 21 health professionals working with participating patients in the PCU. The minimum age of the people interviewed was 26 years old and the maximum age was 94 years old. The professionals most present in the daily life of the unit were undoubtedly the nurses, doctors, and operational assistants.

The characterization of a phenomenon implies identifying the base structure or set of conditions in which the meaning of actions and events occurs, these being fundamental determinants to understand its nature.^11^ The context, despite not determining the experience or fixing the action ‘identifies the sets of conditions in which the problems and/or situations respond to them through some forms of action/interaction and emotions (process) and in doing so generate consequences that, in turn, can recur and impact conditions’.^17^

**Figure 2.**
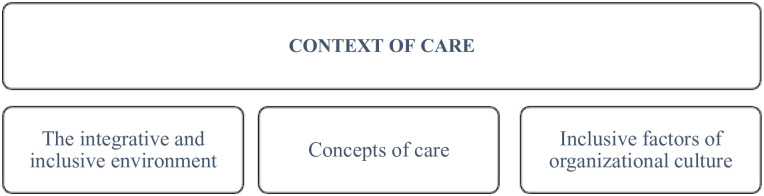
Care context: domains.

Figure 2 expresses the relationship between the factors that shape the Care Context in the PCU studied, which constitute the three domains of this theme, specifically: the integrative and inclusive environment, the concepts of care and the inclusive factors of organizational culture.

The professional care environment is understood as the internal environment existing between members of groups that interact in the context of action and which is deeply associated with the level of motivation, satisfaction, and collaboration between those involved. This environment is affected individually and collectively.^18^

**Figure 3.**
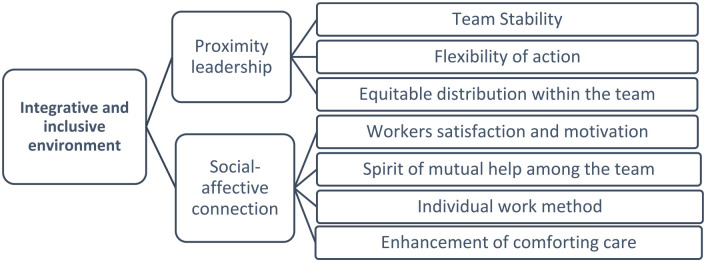
Integrative and inclusive environment: categories and subcategories.

We found that person/environment interactivity created action, where practices and meanings intersected in everyday work, but also in culture, supported by the perspectives of Orem^19^ and Leininger.^17^ After the preparation previously described, which allowed us a sustained insertion into the research field, the observation process began. During this process, we were able to prove the existence *of an integrative and inclusive environment* that emerges as one of the domains of this study. This environment is perceived as the socio-professional and affective environment that is experienced in the service and which contributes to the integration and satisfaction of care actors, being, naturally, enhanced by close leadership and socio-affective-relational connection between the members of the multidisciplinary team, constituting two categories as shown in Figure 3:

Proximity leadership is decisive so that there can be unity and cohesion in the team and also inducing stability, not only in the nursing team, but in the entire multidisciplinary professionals. From the various observations, it was possible to see that the nurse manager has a very present role at all times, from shift changes, to day-to-day work and also in informal situations. There is leadership centered on nurses, with participatory and flexible management, focused on empowering nurses by taking into account the suggestions and opinions given by them. It denotes an attitude of flexibility in action and proximity of the nurse manager, which promotes an emotional connection between the members of the multidisciplinary team, since all members feel that the interest shown by the nurse manager is authentic and genuine. This posture and affection that the leadership promotes generates security that also extends to people admitted to the UCP and their families.

In general, scientific evidence has demonstrated that high levels of professional satisfaction and motivation in the workplace increase work performance. Human beings are complex in their functioning and, therefore, difficult to manage as they differ in skills, abilities, motivations. and preferences. Motivation and satisfaction enhance the spirit of mutual help among the team, as mentioned by the nurses: ‘Everyone plays their role and works as a team. Communication is, as a general rule, very good between all team members. Decisions are made with everyone’s input’ (NI1). This cooperation serves as a basis for demonstrating respect among the team. The idea of cooperation and mutual help at the UCP extends not only to the nursing team but also to other professionals, particularly members of the medical team, ‘(. . .) There are no taboos, there are no egos. There is a team. Everyone is a transmitter and everyone is a receiver’ (PHI1) and also by operational assistants. On the other hand, the working method used by nurses in this unit simultaneously enhances the individualization of care and the accountability of nurses, allowing greater availability for the person at the end of life and their family. This is the individual work method in which each nurse is responsible for providing total care to the patients assigned to them, from hygiene care, nutrition, preparation and administration of therapy, exams, emotional support, clarifying doubts with the family, among others. It is common for the team to try to get nurses to keep the same patients assigned to shifts that are carried out subsequently so that they can maintain the continuity of the care they started and create deeper and more solid connections with both the person and the family. Thus, we observed, in this specific UCP environment, that there were habits and routines that facilitate the organization and management of comfort care.

**Figure 4.**
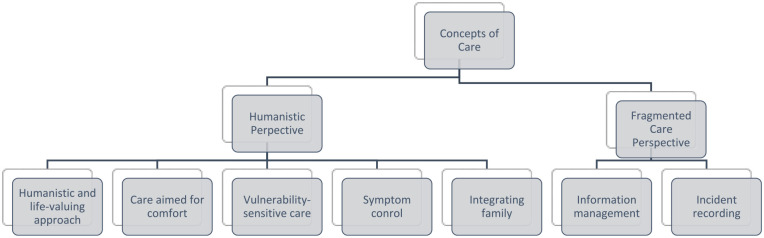
Concepts of care: categories and subcategories.

A concept of care is something that is developed through experience, sensitivity, openness to others, with training representing a crucial role in the development of this same conception. In this study, we focused on care for people at the end of life. PC allows nurses to look at life from a new perspective: caring for and promoting a dignified death for every individual at the end of their life. This care, at the end of life, invites us, in fact, to be able to deal with the incurable disease, with the complexity of the symptoms and the effects of the disease on the person and their family, reflecting on our own fears, incapacities, and limitations. As health professionals, it is also essential to be able to provide care in order to promote quality of life for people who need to be protected and loved while facing an incurable, evolving, but stable disease. Therefore, there is a need for a specific way of caring. Several authors have studied the concepts of end-of-life care and, in general, these concepts are based on the following premises: quality of life, humanistic approach and appreciation of life, control and relief of pain and other symptoms, ethical issues, multidisciplinary approach, the priority of care over healing, comfort, and spirituality.^19,20^ Thus, *concepts of care* emerge as a domain of the care context, which after analyzing the data, we divided into two categories: Humanistic Perspective and Fragmented Care Perspective. From each category, subcategories emerged that are closely linked, being, in most cases, interdependent, as explained in Figure 4:

Regarding the **humanistic life-valuing approach**, we know that this is the essence of PC, approaching it in a human way, valuing and estimating the person for who they were, for their memories, for their life story, but also for what they still are and what they can achieve. Also in the interviews with professionals, it was possible to verify that the person is understood as unique, with biopsychosocial and spiritual dimensions, carrying a history and life experience, with beliefs, and habits, that are valued by healthcare professionals: ‘Comfort is something personal that we have to adapt’ (NI4); the appreciation of life and personal history is very present in all the team’s actions: ‘If we take away from someone at the end of their life all their habits and values (in addition to those that inevitably have to cease to exist) we are contributing even more so that the person feels that they no longer have a place in this world, that they are no longer themselves’ (EE2). There are several authors who corroborate these premises, as different studies prove that, effectively, in a person-centered logic of care, nurses act toward achieving therapeutic objectives, including comfort.^7,19,21,22^

We observed the development care context that aimed to preserve the person’s dignity, with objective aimed at comfort, meeting the person’s multiple needs, also considering other problems, conflicts or ethical dilemmas that could emerge. During the observation period, there were several examples taken from excerpts of the field journal that corroborate this logic aimed at comfort and well-being: ‘(. . .) the patient in question states that she feels uncomfortable being in a single room. She doesn’t like being alone, she feels loneliness as something negative. The nurse in charge quickly came up with a solution so that the patient could move to a double room’ (FJ), ‘(. . .) I entered the room and heard the radio. Classical music and fado (the patient’s favorites)’ (FJ).

In this context, we also observed a **vulnerability-sensitive care perspective**. Vulnerability is a constitutive characteristic of every human being which calls for care. In the specific case of the study context, it was possible in several situations to perceive that people felt vulnerable and that care was strongly sensitive to this vulnerability associated with the end of life: ‘In general, I feel quite vulnerable and I need help. Help to find a way to overcome this. Not to cure myself, I know, but to overcome this’ (PI14). One of the potentialities of the concept of vulnerability in health is the possibility of knowing better the disease and interventions capable of modifying the health-disease situation, with a view to overcoming the biological and behavioral approach.^22–24^ Also, the power of organizing time is often lost, which inevitably causes great suffering. The loss of pleasant daily habits and cultural and routine acts increases this vulnerability even further: ‘I’m devastated, I miss sitting at the table and having a proper meal. I love cooking, it’s part of my culture. I feel bad and I just want to disappear’ (PI11).

Healthcare professionals are attentive to signs of vulnerability, ‘It manifests itself through a vacant look, watery eyes and verbal and non-verbal reports of sadness’ (NI3), ‘Extreme agitation’ (NI8). On the other hand, patients themselves and their families show signs of the same vulnerability typical of the situation experienced in the context of the action: ‘When I was diagnosed with cancer I was completely shocked. I was 39 years old when I was diagnosed with glioblastoma. It was very painful to lose my hair, I’m very vain, as you’ve already noticed. It doesn’t do me any good to be angry, it only makes me worse’ (PI2).

Also the team focuses heavily on **symptom control**, as the structural basis of care. PC must focus on action based on preventing and anticipating crises, controlling the different symptoms caused by the disease or medication, promoting the person’s quality of life until their death, supporting their family in the death process and mourning, as well as offering moral, relational, spiritual, and religious support to the person and family^3^: ‘The nurse concerned about the discomfort that aspiration caused to the patient chose to aspirate only once, and then administered subcutaneous medication (Buscopan) to keep the patient more comfortable. The mouth was also cleaned with water and the mucous membranes were moistened’ (FJ). At the same time, the team’s concern with all types of symptoms that may cause suffering is perceived by the patient and their family and has a great impact on their quality of life: ‘I was comforted because I was pain free and I felt a lot better, I felt like I was in the right place, that I wasn’t alone and deep down that I was better here than at home’ (PI5).

The **integration of the family** in care enhances their capacity to respond to the needs of the person at the end of life and, when the family is available, promotes feelings of satisfaction, in an integrative environment. Integration requires a space and time that allows the creation of complicity with the family and simultaneously allows the family to understand the dynamics of the unit, in order to participate more actively. The family also considers that they comfort their loved one in ways that professionals could not: ‘My way of comforting is to come here to be with her, to keep her company. We pray together and ask God that she doesn’t suffer until it is time for her to go. We sing. (. . .) and I’ll tell her some gossip’ (FAMI14); ‘Being present, keeping company, talking about everyday life. Telling things about the house, the children, the grandson. . . Bring him a little bit of our home, of our life (. . .), to take him away from this world of illness, fear and darkness’ (FAMI18). Family members feel included in the care process; they feel their actions and care interventions are useful and cause direct benefit to the patient: ‘We couldn’t take away his pain, but we can take away the pain of being alone’ (FAMI8). The family feels involved and feels like they’re the target of the team’s attention, interest, and concern: ‘They health team talks to him (the patient) and to me like no one else does. They organized the arrival of my niece, something that was fundamental for my brother’s peace of mind’ (FAMI9).

Although the humanistic perspective is dominant in the context of PC, the social model assumes a ‘Fragmented’ Care Perspective based on a biomedical model that appears as a second category. This fragmentation is particularly significant in aspects relating to information management and the recording of occurrences that emerged as subcategories. As we know, the nursing process is a systematic method of organizing nursing decision making and problem solving when planning and providing care to people. As an indispensable tool for nurses’ daily lives, the nursing process allows you to plan and decide on the care you will provide through a dynamic and individualized process. In the context of action studied, in some situations, we were able to observe gaps regarding the recording of occurrences relating to comfort in the various phases of the nursing process.

With regard to **information management**, we know that records/notes must be objective, accurate, complete, concise, updated, and organized. Notes can also be very important when it comes to applying the problem-solving method that involves identifying a problem and all the issues involved. Regarding nurses, information management is particularly important, especially regarding the clinical situation of the person at the end of life, with the nurse’s role as the main link between patient/family/team: ‘Nurses are the main bridge between all professionals in the unit, patients and families. They are a huge asset’ (PHI 2).

With regard to **incident recording**, we know that information and its implementation are of great importance for nursing. The registration itself proposes to improve communication between everyone who cares for the person, providing common and comprehensive attention to needs. However, in the context of the action, we noticed that nursing notes are not very expressive regarding the topic of comfort, whether in the initial assessment, diagnosis, interventions, and even in the final assessment phase. It is not always easy to document the various interventions carried out, as we found during the observation and were able to record in the field journal: ‘Regarding records: I note that there is no specific area where comfort needs or comforting interventions made to patients are recorded. All the notes I observed were made by nurses. In the medical records, I did not find any record regarding aspects related to comfort, other than with regard to pain control and relief’ (FJ).

**Figure 5.**
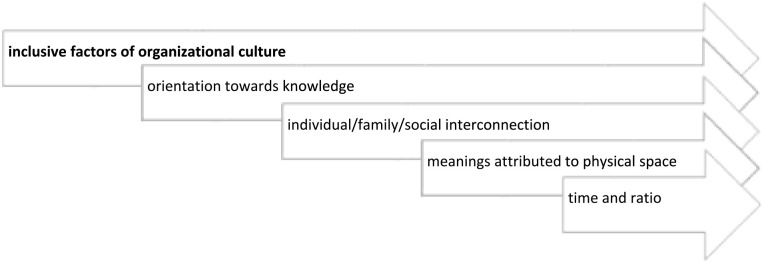
Characterization of the organizational culture of care at the palliative care unit: domain and categories.

Regarding the *inclusive factors of organizational culture*, we understand that comfort is shaped by the organizational culture of the unit as well as by the relationships between the multidisciplinary team. These relationships generate a symbolic and cultural world, in a process of secondary socialization and structuring construction, in the personal and professional development of each of the actors. The processes that allow nursing care to be produced and the relationships between actors mirrors this culture. Nurses, as managers of this process of reflexivity, are central to this organizational culture, in a project that is collective and integrated into a broader relationship of providing care to people at the end of life and their family. In this domain, five categories emerged: orientation toward knowledge; individual/family/social interconnection; meanings attributed to physical space; time and ratio; reflexivity and analysis culture (Figure 5).

The unit under study, within its geographical limits, self-manages and articulates with its external environment, both with regard to other services of the health organization, and with all community support services. There is a strong *orientation toward knowledge* in which the team is concerned with keeping their knowledge up to date as well as the existence of various internal training on the unit. At the same time, the team (mainly physicians and nurses) seeks to receive some external training in the end-of-life area with the clear objective of keeping their knowledge aligned with the latest guidelines for PC: ‘almost all health professionals working here have training in palliative care. They really like being here, it’s where they belong’ (PCAI5). On the other hand, we clearly assess attitudes associated with the understanding of *individual/family/social interconnection*, in the relational development of service and care. This individual/family and social interconnection is highly valued and is deeply rooted in the organizational culture of this unit, as we saw in the field diary observations: ‘Family is present throughout the day since yesterday. Today the wife will spend the night so that, if the patient dies, she can be present. When the patient was lucid, he expressed that he did not want to die alone. (. . .) Specifically, he would like to be with his wife, who had been his lifelong companion’ (FJ).

This organizational culture in particular allows family visits to be extended, which inevitably proves to be very comforting for the majority of patients: ‘Families can spend the night here, be here all day. In other places it is not allowed, this is a great advantage’ (PCAI3).

With regard to the organization’s culture, there is also a strong sensitivity to the *meanings attributed to physical space*, with the concern for the environment being very evident: ‘I notice that there is a concern regarding the physical environment in comfort care’ (FJ); ‘(. . .) it is explained that you can bring your own clothes and that the rooms were designed to promote tranquility and comfort, therefore they have the following amenities: articulated bed, bedside table and side table, chaise longue for the patient and for visitors staying overnight, a built-in personal locker, television, individual sanitary facilities equipped with a support system for the disabled and an emergency alarm system’ (FJ). We can verify this cultural conception in several moments of the interviews with the health team, who recognize that this particular unit and its organizational culture allows the task of comforting the person to be much more accessible: ‘This unit is organized in such a way that there are spaces for patients to have leisure time/meals’ (PCAI5); ‘Then the fact that most of the rooms are individual allows you to be more comfortable in your privacy or with your family. You can cry’ (PCAI5). In addition, several team members also draw attention to the fact that there is a dedicated area for a more complete and comfortable shower, in a specific room with a heater, music and even, scented candles: ‘The existence of a bathroom with a bath stretcher, which allows users with a high degree of dependence to have hygiene care using a shower’ (NI9). ‘It’s really great for patients who have been bathing in bed with just a sponge for several days or weeks’ (PCAI7). Still regarding the physical space, the team considers that the dining/meeting room is an extremely important space with several functions; however, it can and often functions as a social space between family and patient’s usual: ‘The meeting room to meet with the family’; ‘We have our own library with several books on different themes’ (NI4).

**Time and ratio** are also mentioned as important factors: ‘This unit is small and we can reach the patients, be close to them, it is very different from other places, we have TIME’ (PCAI2); ‘It’s easier to comfort here. We have fewer patients, much more time for them’ (PCAI4).

The context of action is characterized by a culture of reflexivity and analysis of the actors’ experiences. It is common to reflect on individual and collective attitudes, which ends up leading to frequent changes in ideas, attitudes, procedures, and interventions, thus demonstrating a culture that is open to change within the team, whenever this change is beneficial to all those involved in the context of the action. In general, all actors involved recognize that the context of action is built on very specific characteristics that make it distinct from other environments and other organizational cultures: ‘The organization understands comfort care as indispensable, indeed. Everyone works towards this and the organization itself encourages comfort to be taken into account in several aspects: time, physical space, training of health professionals’ (NI3); ‘This unit was built to promote comfort, it was built with patients, families and professionals in mind. There is a culture of comfort on this place’ (NI2).

From the collected data, we understand that the entire organizational culture seems designed to converge in a single direction: meeting the comfort needs of the person at the end of life.

## Limitations of the study

Most of the data collection took place during the pandemic period, which created several obstacles, namely, the creation of a focus group, which was not possible, due to the need to reduce the risks of contamination at that time.

However, knowing that each ethnographic study cannot be ‘reproduced’, these results can serve as a guide for building and consolidating knowledge about a phenomenon considered noble and associated with nurses’ interventions.

## Conclusion

In this article, we addressed the context of care, the psychical space from the idealized to the real, where objects and provisions support the construction of the comfort process as an entity that integrates culture, mediating established conceptions of care and allowing the deepening of knowledge. We also addressed the integrating factors of organizational culture that structure the culture of the context as well as its importance as a distinctive factor of this unit recognized by all the actors.

Interviews and the observation in the field were coded as follows: PI – Patient Interview; PHI – Physician Interview; FAMI – Family Interview; PSI – Psychologist Interview; NTI – Nutritionist Interview; PCAI – Patient Care Assistant Interview; FJ – Field Journal; NI – Nurse Interview.

## Supplemental Material

sj-pdf-1-pcr-10.1177_26323524241258781 – Supplemental material for The context of care as a supporting axis for comfort in a palliative care unit Supplemental material, sj-pdf-1-pcr-10.1177_26323524241258781 for The context of care as a supporting axis for comfort in a palliative care unit by Raquel Alexandra Machado Pereira and Patrícia Cruz Pontífice Sousa Valente Ribeiro in Palliative Care and Social Practice

## Declarations

**Ethics approval and consent to participate:** The study was conducted according to the guidelines of the Declaration of Helsinki and approved by the CHBM Ethics Committee and Universidade Católica Portuguesa de Lisboa Ethics Committee. Approval number or ID provided by the CHBM Ethics Committee and Universidade Católica Portuguesa de Lisboa Ethics Committee: Memorando Nota Interna: 48/2019.

**Consent for publication:** Participants provided written informed consent prior to the start of data collection.

**Availability of data and materials:** Ethical approval precludes the data being used for another purpose or being provided to researchers who have not signed the appropriate confidentiality agreement. Specifically, the ethical approval specifies that all results are in aggregate form to maintain confidentiality and privacy and precludes individual level data being made publicly available. All aggregate data for this study are freely available and included in the article. Interested and qualified researchers may send requests for additional data to Raquel Pereira at pereira.raquel12@gmail.com.

## References

[1] Pinto S Fumincelli L Mazzo A , et al. Comfort, well-being and quality of life: discussion of the differences and similarities among the concepts. Porto Biomed J 2017; 2: 6–12. 32258577 10.1016/j.pbj.2016.11.003 PMC6806988

[2] Tsai J-L , et al. Comfort: a concept analysis. Hu li za zhi J Nurs 2021; 59: 77–82. 22314653

[3] ACSS – Departamento de Gestão da Rede de Serviços e Recursos em Saúde (DRS). Relatório de monitorização da Rede Nacional de Cuidados Continuados Integrados (RNCCI) – 2015. 2016.

[4] Capelas ML Coelho S Silva S , et al. (2017). Cuidar a pessoa que sofre – uma teoria de cuidados paliativos. Universidade Católica Portuguesa Editora. pp. 69–70.

[5] Doyle D Jeffrey D . Palliative care in the home. Oxford: Oxford University Press, 2005.

[6] Neto IG . Princípios do Controlo de Sintomas, um pilar dos Cuidados Paliativos. Revista da Ordem dos Médicos (Edição Online), https://ordemdosmedicos.pt/principios-do-controlo-de-sintomas-um-pilar-dos-cuidados-paliativos-por-isabel-galrica-net (2017).

[7] Radbrunch L De Lima L Knaul F , et al. Redefining palliative care – a new consensus – based definition. J Pain Symptom Manage 2020; 60: 754–764. 32387576 10.1016/j.jpainsymman.2020.04.027 PMC8096724

[8] Sousa P . O conforto da pessoa idosa. 2ª ed. Lisboa: Universidade Católica Editora, 2020.

[9] Serrano MT . Desenvolvimento de competências dos enfermeiros em contexto de trabalho. Tese de doutoramento, Universidade de Aveiro, Repositório da Universidade de Aveiro, 2008, http://hdl.handle.net/10773/1479

[10] Leininger M . Qualitative research methods in nursing. New York, NY: Grue & Strotton, 1985.

[11] Spradley JP . The ethnographic interview. Long Grove, IL: Waveland Press, Inc., 2016.

[12] Spradley JP . Participant observation. Long Grove, IL: Waveland Press, Inc., 2016.

[13] Tong A Sainsbury P Craig J . Consolidated criteria for reporting qualitative research (COREQ): a 32-item checklist for interviews and focus groups. Int J Qual Health Care 2007; 19: 349–357. 17872937 10.1093/intqhc/mzm042

[14] Bogdan Bilklen RS . Investigação qualitativa em educação: uma introdução à teoria e aos métodos. 2nd ed. Porto: Porto Editora, 2013.

[15] Creswell JW . Investigação qualitativa e projeto de pesquisa: escolhendo entre cinco abordagens. 3rd ed. Porto Alegre: Penso Editora, 2014.

[16] Johnson JL Adkins D Chauvin S . A review of the quality indicators of rigor in qualitative research. Am J Pharm Educ 2020; 84: 7120. 32292186 10.5688/ajpe7120 PMC7055404

[17] Leininger M . Culture care diversity and universality theory and evolution of the ethnonursing method. In: Leininger M McFarland M (eds.) Culture care diversity and universality: a worldwide nursing theory. 2nd ed. Sudbury, MA: Jones and Bartlett Publishers, 2006, pp. 1–41.

[18] Corbin J Strauss A . Basics of qualitative research. Thousand Oaks, CA: Sage, 2008, pp. 80–91.

[19] Orem D . Nursing: concepts of practice. 6ª ed. Saint Louis, MO: Mosby, 2001.

[20] Carvalhal R . Relação enfermeira/idoso/família: Construção, desenvolvimento e prática. Tese de Doutoramento em Enfermagem, Universidade de Lisboa, Repositório da Universidade de Lisboa, 2012, pp. 149–159, http://hdl.handle.net/10451/7349 (last accessed 03 May 2022).

[21] Alves R Melo M Andrade S , et al. Saberes e práticas sobre cuidados paliativos segundo psicólogos atuantes em hospitais públicos. Psicologia, Saúde & Doenças 2014; 15: 78–96.

[22] Apóstolo J . O Conforto nas Teorias de Enfermagem – Análise do Conceito e Significados Teóricos. Coimbra: Referência, 2009, pp. 61–67

[23] Kolcaba K . Comfort. In: S Peterson T Bredow (Orgs). Middle range theories. Application to nursing research. 3ª ed. Wolters Kluwer Health | Lippincott Williams & Wilkins, 2013, pp. 193–207.

[24] Oliveira L Almeida M Silva C , et al. Aspetos éticos do cuidado de enfermagem ao idoso em cuidados paliativos. Enferm Foco 2021; 12: 393–399.

